# Development of an Improved Rotational Orthosis for Walking With Arm Swing and Active Ankle Control

**DOI:** 10.3389/fnbot.2020.00017

**Published:** 2020-04-22

**Authors:** Zaile Mu, Qiuju Zhang, Guo-Yuan Yang, Le Xie, Juan Fang

**Affiliations:** ^1^School of Mechanical Engineering, Jiangnan University, Wuxi, China; ^2^Med-X Research Institute and School of Biomedical Engineering, Shanghai Jiao Tong University, Shanghai, China; ^3^Institute of Medical Robotics, Shanghai Jiao Tong University, Shanghai, China

**Keywords:** interlimb neural coupling, adjustable admittance control, pole-placement design, active ankle control, gait rehabilitation

## Abstract

Based on interlimb neural coupling, gait robotic systems should produce walking-like movement in both upper and lower limbs for effective walking restoration. Two orthoses were previously designed in our lab to provide passive walking with arm swing. However, an active system for walking with arm swing is desirable to serve as a testbed for investigation of interlimb neural coupling in response to voluntary input. Given the important function of the ankle joint during normal walking, this work aimed to develop an improved rotational orthosis for walking with arm swing, which is called ROWAS II, and especially to develop and evaluate the algorithms for active ankle control. After description of the mechanical structure and control schemes of the overall ROWAS II system, the closed-loop position control and adjustable admittance control algorithms were firstly deduced, then simulated in Matlab/Simulink and finally implemented in the ROWAS II system. Six able-bodied participants were recruited to use the ROWAS II system in passive mode, and then to estimate the active ankle mechanism. It was showed that the closed-loop position control algorithms enabled the ROWAS II system to track the target arm-leg walking movement patterns well in passive mode, with the tracking error of each joint <0.7°. The adjustable admittance control algorithms enabled the participants to voluntarily adjust the ankle movement by exerting various active force. Higher admittance gains enabled the participants to more easily adjust the movement trajectory of the ankle mechanism. The ROWAS II system is technically feasible to produce walking-like movement in the bilateral upper and lower limbs in passive mode, and the ankle mechanism has technical potential to provide various active ankle training during gait rehabilitation. This novel ROWAS II system can serve as a testbed for further investigation of interlimb neural coupling in response to voluntary ankle movement and is technically feasible to provide a new training paradigm of walking with arm swing and active ankle control.

## Introduction

In parallel with the traditional therapeutic techniques for walking rehabilitation, robot-assisted gait training reduces the working load of therapists and shortens the rehabilitation cycle for patients (Lum et al., 2002). Rehabilitation robotic systems can provide repetitive and intensive training in various modalities (Orrell et al., 2006; Muramatsu and Takano, 2007; Chang and Kim, 2013). Accordingly, they are widely used clinically to assist rehabilitation of walking.

Human walking is a synchronous movement of upper and lower limbs. Arm swing helps to stabilize gait and to reduce energy expenditure (Bruijn et al., 2010). The coordinated movement between upper and lower limbs during human walking is considered to be neutrally coordinated (Dietz et al., 2010). Based on the interlimb neural coupling, gait robotic systems should produce synchronous movement of upper and lower limbs for effective walking restoration (Ferris et al., 2006). Two rotational orthoses for walking with arm swing were designed in our lab, called ROWAS (Fang et al., 2017a) and aROWAS (Fang et al., 2017b). The drives were installed bilaterally on the shoulder, hip, knee, and ankle joints. Both ROWAS and aROWAS achieved walking-like coordinated movement in the bilateral upper and lower limbs in passive mode. However, an active system for arm-leg walking is desirable to serve as a testbed for investigation of interlimb neural coupling in response to voluntary input.

Rehabilitation robotic systems often have passive and active movement modes to provide various training modalities for patients with different motor functions. Passive training, which allows patients to follow a fixed reference trajectory, is suitable for those in the early post-injury stage. Cai et al. observed that the spinal-cord-transected mice that performed assist-as-needed training regained better movement abilities than the mice that performed training with fixed trajectories (Cai et al., 2006). Active training is often used by the patients who can perform certain voluntary movement (Tian et al., 2007). Furthermore, the ankle joint plays a key role in human walking (Kepple et al., 1997; Mcnealy and Gard, 2008), such as swing initiation, weight support and forward progression. So active training of the ankle joint is essential for gait restoration for the patients with impaired walking ability. However, there are few gait rehabilitation robotics in the market that integrate active ankle training (Pratt, 2000; Susanna et al., 2008; Esquenazi and Packel, 2012).

There are several control approaches, such as the state-space method in modern control (Rubio et al., 2019a), and its successful application in electric vehicle control (Gao et al., 2020), perturbations attenuation (Rubio et al., 2019b) and manipulator control (Rubio, 2018). Admittance algorithms in classical control are often used and successfully applied in many rehabilitation systems for active training. Compared with impedance control (Hogan, 1985), admittance control does not require an accurate plant model, and can be implemented by adding a force sensor on the link position (Richardson et al., 2003). Richardson et al. applied an admittance scheme to achieve a flexible control of a pneumatic physiotherapy robotic system (Richardson et al., 2003). Saglia et al. developed admittance control algorithms for active ankle rehabilitation (Saglia et al., 2010). Taherifar et al. designed a smart assist-as-needed control system with a variable admittance control strategy which reduced the interaction energy between the user and the exoskeleton device (Taherifar et al., 2018). Zhang et al. developed an admittance controller with EMG-based torque prediction and achieved multiple assistive gait patterns (Gui et al., 2017). Admittance control algorithms promote active participation of the users during training in the rehabilitation robotic systems.

Our previous ROWAS and aROWAS systems only provided passive training (Fang et al., 2017a,b). Given the important function of the ankle joint during normal walking, it is desirable to implement active ankle movement training in the arm-leg synchronized walking. Furthermore, such a robotic system can serve as an effective tool for investigation of interlimb neural coupling in response to voluntary input. This work aimed to develop an improved rotational orthosis for walking with arm swing, which is called ROWAS II, and especially to develop and evaluate the algorithms for active ankle control. Our previous work briefly described the admittance control algorithms which produced active ankle movement with a fixed admittance gain (Mu et al., 2019). This work explained this investigation in detail by firstly assessing the functionality of the overall ROWAS II system, and then evaluating the adjustable admittance control algorithms for the ankle mechanism in the ROWAS II system.

## Methods

The mechanical development of the ROWAS II system was performed using SolidWorks (Version 2016, Dassault Systèmes SolidWorks Corporation, Massachusetts, USA). After the overall control scheme of the ROWAS II system was described, the control algorithms for passive and active training were firstly deduced, then simulated in Matlab/Simulink (MathWorks Inc., 2015a, USA) and finally implemented in the ROWAS II system. Six able-bodied participants were recruited to evaluate the functionality of the ROWAS II system and especially the active ankle mechanism.

### Mechanical Development

**Figure 1:**
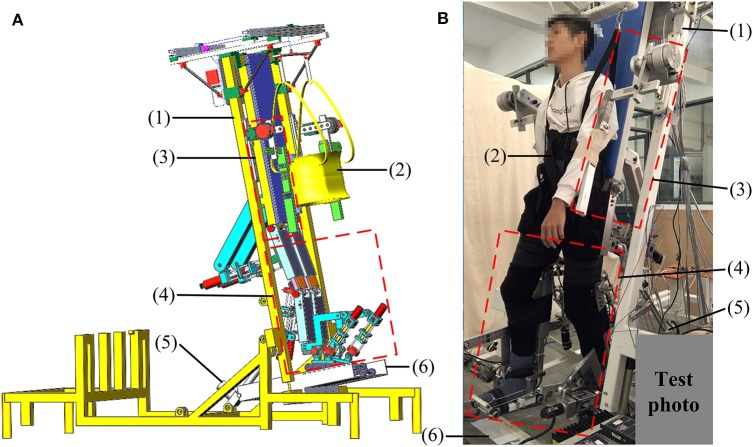
The ROWAS II system: **(A)** the CAD model and **(B)** the prototype with a test participant. (1) Bed frame, (2) BWS system, (3) mechanism for the upper limb, (4) mechanisms for the lower limbs, (5) linear actuator, and (6) ground-simulation plate. Adapted from our previous paper (Mu et al., 2019) with permission (license:4797140179422) from IEEE.

The ROWAS II system is mainly composed of a bed frame, a body weight support (BWS) system, mechanisms for the upper and lower limbs and a ground-simulation plate (Figure 1). The bed frame is composed of square steels, while the mechanisms for the upper and lower limbs are made of aluminum alloy. Using two linear actuators (1013CPC, Moteck Co., Ltd., China), the bed frame can be tilted from a horizontal position to a vertical position to provide training in different positions. Actuated by a stepper motor (86HS45, Leadshine Technology Co., Ltd., China) with a gearbox (ratio of 10:1), the BWS system can support a user of 100 kg. In addition, the system can adjust the lengths of the upper and lower limbs so as to fit users with different heights between 1.50 and 1.80 m. The mechanical stops constrain the mechanical range of motion (ROM) for each joint. The emergency stop button for the control system can ensure users' safety in the ROWAS II system.

**Figure 2:**
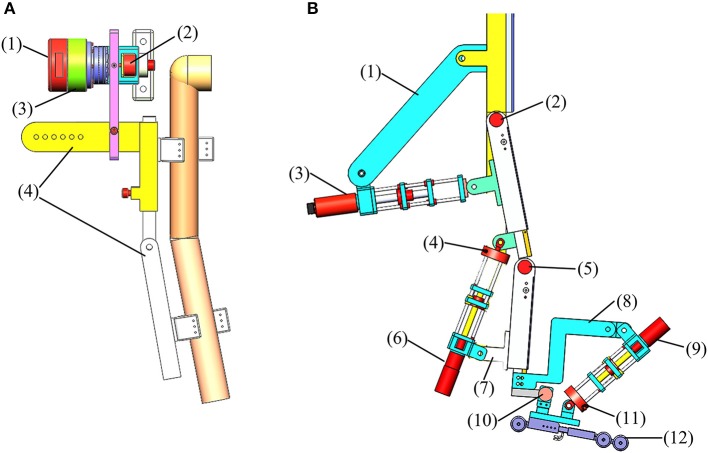
The models of the mechanisms for the right upper and lower limbs. **(A)** Upper limb model: (1) shoulder motor (EC 90), (2) angle sensor, (3) gearbox (ratio of 100:1), and (4) shoulder adjusting bars. **(B)** Lower limb model: (1) hip assisting bar, (2), (5) and (10) angle sensor, (3) hip motor (RE 50), (4) and (11) force sensor, (6) knee motor (RE 40) with a gearbox (ratio of 3.5:1), (7) knee assisting bar, (8) ankle assisting bar, (9) ankle motor (RE 40), and (12) shoe platform.

The mechanisms of the upper and lower limbs (Figure 2) were actuated by servo drives (maxon motor, Switzerland). Different from the previous ROWAS systems which had the motors mounted on bilateral sides of the hip, knee, and ankle joints (Fang et al., 2017a,b), the ROWAS II system used series linear actuators, which were installed behind the hip and knee mechanisms and in front of the ankle mechanism. The series linear actuator composed of a motor and a ball screw assembly (TBI Motion Technology Co., Ltd., China). It retracted or extended to actuate the hip, knee, and ankle joints. The force sensor (Jnsensor Co., Ltd., China) at the end of the actuator can measure the force to pull and push the leg segment.

### Control Development

#### Overall Control Scheme

**Figure 3:**
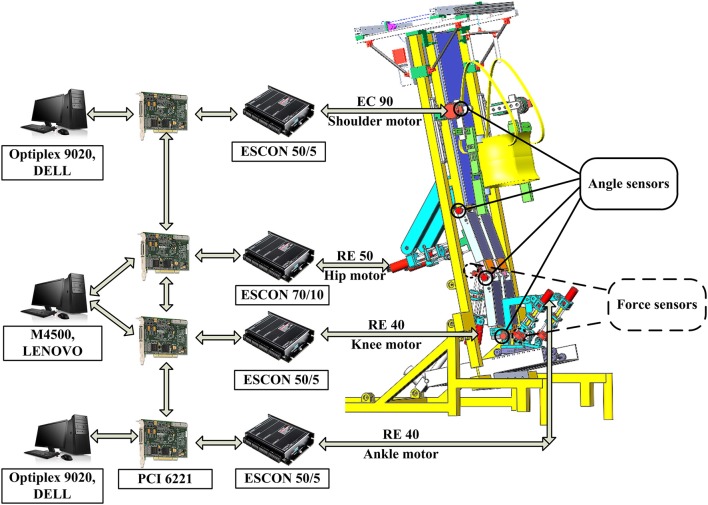
Control scheme.

To control the eight motors for synchronized movement in the bilateral upper and lower limbs, eight hardware controllers (maxon motor, Switzerland) were used and set in speed mode (Figure 3). As one NI data acquisition card (PCI 6221, National Instruments, USA) has only two analog-output channels, one PCI card can transfer data for two hardware controllers. Therefore, four PCI cards were used for the eight hardware controllers and were inserted into three computers. One computer [Lenovo (Beijing) Co., Ltd., China] has two PCI slots while the other two computers [Dell (China) Co., Ltd., China] have one PCI slot. These three computers established synchronization via a digital trigger sent by these PCI cards. The angle sensors (Xinle Co., Ltd., Shanghai, China) measured the movement of the eight joints, which was used to develop the closed-loop position control algorithms. The force sensors provided the force information from the participants, which was used to develop the adjustable admittance control algorithms. Part of the following control algorithms was adapted from our previous paper (Mu et al., 2019) with permission (license:4797130595869) from IEEE.

#### Closed-Loop Position Control for the ROWAS II System

**Figure 4:**
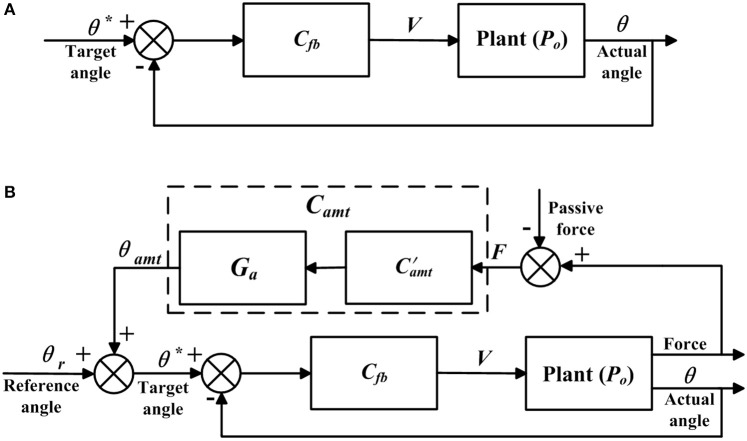
Control algorithms. Adapted from our previous paper (Mu et al., 2019) with permission (license:4797130595869) from IEEE. **(A)** Closed-loop position control and **(B)** adjustable admittance control.

The closed-loop position control algorithms were developed to make the actual angle θ track the target angle θ^*^ (Figure 4A), and were implemented in the bilateral mechanisms of the shoulder, hip, knee, and ankle joints to enable passive walking-like training in the ROWAS II system.

The nominal plant *P*_*o*_(*s*) linking the input voltage *V* to the hardware controller and the actual angle, was expressed as:

(1) $$\begin{array}{l} V\to \theta \;:\;P_{\text{o}}\left(s\right)=\frac{B_{\text{o}}\left(s\right)}{A_{\text{o}}\left(s\right)}=\frac{k}{s},n_{a}=1, \end{array}$$

where *n*_*a*_ is the degree of the polynomial *A*_*o*_(*s*), and the steady-state gain *k* was obtained empirically using system identification. The feedback controller *C*_*fb*_*(s)* was obtained using the pole-placement approach (Aström and Murray, 2010), which was a linear, time invariant and strictly-proper transfer function,

(2) $$\begin{array}{l} \theta^{\text{*}}\text{-}\theta \to V:\;C_{fb}\left(s\right)=\frac{G\left(s\right)}{H\left(s\right)}, \end{array}$$

where the polynomials *G*(*s*) and *H*(*s*) can be identified as

(3) $$\begin{array}{l} G\left(s\right)=g_{n_{g}}s^{n_{g}}+g_{n_{g}-1}s^{n_{g}-1}+\cdots +g_{0}, \end{array}$$

(4) $$\begin{array}{l} H\left(s\right)=s^{n_{h}}+h_{n_{h}-1}s^{n_{h}-1}+\cdots +h_{0}. \end{array}$$

The transfer function of the overall system *T*_*o*_(*s*) is

(5) $$\begin{array}{l} \theta^{\text{*}}\to \theta :T_{o}\left(s\right)=\frac{C_{fb}P_{o}}{1+C_{fb}P_{o}}=\frac{B_{o}G}{A_{o}H+B_{o}G}. \end{array}$$

So, the order of *T*_*o*_(*s*) *n*_1_ and the number of the unknown parameters *n*_2_, are expressed as

(6) $$\begin{array}{l} n_{1}=n_{a}+n_{h}, \end{array}$$

(7) $$\begin{array}{l} n_{2}=n_{g}+1+n_{h}, \end{array}$$

To obtain a unique solution to the controller *C*_*fb*_(*s*), *n*_1_ should be equal to *n*_2_, thus,

(8) $$\begin{array}{l} n_{a}=n_{g}+1. \end{array}$$

Equations (1) and (8) yield *n*_g_ = 0. Therefore, the transfer functions of *C*_*fb*_(*s*) and *T*_*o*_(*s*) are:

(9) $$\begin{array}{l} C_{fb}\left(s\right)=\frac{G\left(s\right)}{H\left(s\right)}\text{=}\frac{g_{\text{0}}}{s+h_{\text{0}}}, \end{array}$$

(10) $$\begin{array}{l} T_{\text{o}}\left(s\right)=\frac{C_{fb}P_{\text{o}}}{1+C_{fb}P_{\text{o}}}=\frac{kg_{\text{0}}}{s^{2}+h_{0}s+kg_{0}}. \end{array}$$

The relationship between the target angle θ^*^ to the actual angle θ of the overall system in the time domain is:

(11) $$\begin{array}{l} \theta^{\text{*}}=\frac{\ddot{\theta }+h_{\text{0}}\dot{\theta }+kg_{\text{0}}\theta }{kg_{\text{0}}}. \end{array}$$

Setting *T*_*o*_(*s*) to a standard 2nd order transfer function

(12) $$\begin{array}{l} T_{\text{o}}\left(s\right)=\frac{\omega_{n}^{2}}{s^{2}+2\xi \omega_{n}s+\omega_{n}^{2}}, \end{array}$$

where ξ and ω_*n*_ are the damping ratio and natural frequency, respectively. Comparison between Equations (10) and (12) results in

(13) $$\begin{array}{l} g_{0}=\frac{\omega_{n}^{2}}{k},h_{0}=2\xi \omega_{n}. \end{array}$$

Therefore, the feedback controller *C*_*fb*_(*s*) is

(14) $$\begin{array}{l} C_{fb}\left(s\right)=\frac{g_{0}}{s+h_{0}}\text{=}\frac{\frac{\omega_{n}^{2}}{k}}{s+2\xi \omega_{n}}. \end{array}$$

In the closed-loop position control algorithms, the critical damping ratio ζ = 1 was employed when the system has no overshoot in response to a step input. In this case, the parameter ω_*n*_ is approximately ω_*n*_ = 3.35*/t*_*r*_ (Nise, 2000), where *t*_*r*_, which is the rise time of the closed-loop system, was defined here as *t*_*r*_ = 0.8 s.

#### Adjustable Admittance Control for the Ankle Joint

The adjustable admittance control algorithms provided various degrees of active movement control, and were implemented in the right ankle mechanism. The adjustable admittance control (Figure 4B) differed from the closed-loop position control (Figure 4A) in that the target position θ^*^ was modified by θ_*amt*_, which depended on the active force *F* (Figure 4B) exerted by the participant and the adjustable admittance gain *G*_*a*_, which are described now. In the closed-loop position control algorithms, the target position θ^*^ was the reference ankle trajectory, while in the adjustable admittance control algorithms, the target position θ^*^ was the reference ankle trajectory plus the admittance angle θ_*amt*_.

The admittance control algorithms used a stiffness-damping-inertial system to represent the relationship between the adjusted angle θ_*amt*_ and active force *F*, giving

(15) $$\begin{array}{l} F=M{\ddot{\theta }}_{amt}+P{\dot{\theta }}_{amt}+Q\theta_{amt}, \end{array}$$

where *M, P*, and *Q* are, respectively, the inertia, damping and stiffness of the admittance control strategy. By Laplace transformation, the admittance controller *C*_*amt*_ was yielded as

(16) $$\begin{array}{l} C_{amt}\left(s\right)=\frac{\theta_{amt}\left(s\right)}{F\left(s\right)}=\frac{1}{Ms^{2}+Ps+Q}. \end{array}$$

Thus

(17) $$\begin{array}{l} C_{amt}\left(s\right)=\frac{1}{Q}\times \frac{\frac{Q}{M}}{s^{2}+\frac{P}{M}s+\frac{Q}{M}}. \end{array}$$

To develop the adjustable admittance control algorithms, an admittance gain *G*_*a*_ was introduced. So

(18) $$C_{amt}(s)=G_{a}C_{\;amt}^{\prime },$$

(19) $$C_{\;amt}^{\prime }(s)=\frac{\frac{Q}{M}}{s^{2}+\frac{P}{M}s+\frac{Q}{M}}.$$

Setting $C_{\;amt}^{\prime }$(*s*) to a standard second-order transfer function as

(20) $$C_{\;amt}^{\prime }(s)=\frac{\omega_{n}^{2}}{s^{2}+2\xi \omega_{n}s+\omega_{n}^{2}},$$

where ξ and ω_*n*_ are the damping ratio and natural frequency respectively. Comparison between Equations (19) and (20) results in

(21) $$\begin{array}{l} \omega_{n}=\sqrt{\frac{Q}{M}},\xi =\frac{P}{2\sqrt{MQ}}. \end{array}$$

Therefore

(22) $$\begin{array}{l} Q=M{\omega_{n}}^{\text{2}},P=2\xi \sqrt{MQ}. \end{array}$$

The mechanical structure of the ankle mechanism yielded the inertial component *M* = 2 *Ns*^2^/°. Using the same parameters ξ = 1, *t*_*r*_ = 0.8 s as adopted in the position control, Equation (22) yielded the admittance parameters *P* = 16.65 *Ns*/°, *Q* = 34.92 *N*/°.

In the admittance control algorithms the admittance gain *G*_*a*_ was tuned. Actuator saturation is often observed in movement control (Sun et al., 2020). Inclusion of saturation functions in the control system can effectively reduce the tracking error brought by input saturation (Sun et al., 2019), and can be also applied in the current study. A saturation block from Matlab/Simulink was included to restrict the motor speed within the limit.

Although the adjustable admittance controller in Equation (20) and the transfer function of the overall position control system *T*_o_(*s*) in Equation (12) coincidently used the same response criteria such as the damping ratio and rise time, which largely depended upon the mechanical setup of the ROWAS II system, the adjustable admittance controller and the position controller were totally different in their functions and physical meanings.

#### Simulation of the Control Algorithms

**Figure 5:**
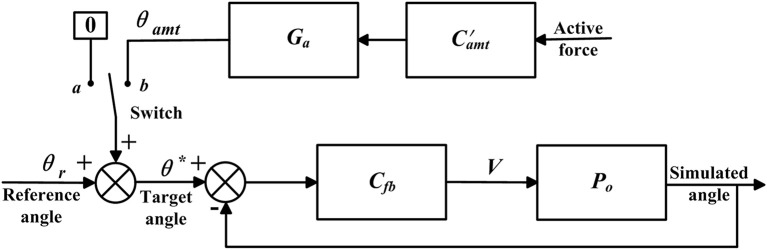
Simulation model.

A simulation model was developed in Matlab/Simulink to evaluate the control algorithms (Figure 5). *P*_*o*_ was the experimentally obtained transfer function, while *C*_*fb*_ and $C_{\;amt}^{\prime }$ were, respectively, the control algorithms deduced previously. The model simulated the closed-loop position control algorithms in passive mode when the switch was connected to *a*. Updating *P*_*o*_ to the transfer functions for the mechanisms of bilateral shoulder, hip, knee, and ankle joints yielded the simulation results of synchronous movement in the overall ROWAS II system running in passive mode. When the switch was connected to *b*, the model simulated the adjustable admittance control algorithms in active mode, where *P*_*o*_ was the transfer function of the right ankle mechanism. The active force was obtained based on the experimental force measurement. The model simulated at a sampling frequency of 200 Hz. The simulated angle was used to calculate the root-mean-square tracking error (RMSE) in section Experimental Evaluation of the ROWAS II System.

### Experimental Evaluation of the ROWAS II System

Six able-bodied participants were recruited to evaluate the system functionality (Table 1). In this experiment, the bed frame was tilted up to 75° relative to the ground, and the walking cycle of the ROWAS II system was 7 s. The sampling frequency of the control system was 200 Hz. Besides, the joints angle profiles for the bilateral shoulder, hip, knee, and ankle joints, which were recorded in a standard normal gait analysis experiment (Fang et al., 2015), were used as the reference trajectories θ_*r*_ for the ROWAS II system.

**Table 1:** Participant information.

**Participant**	**P1**	**P2**	**P3**	**P4**	**P5**	**P6**
Age range (yrs)	20–25	20–25	20–25	25–30	30–35	20–25
Body mass (kg)	55	48	59	62	55	74
Height (m)	1.65	1.60	1.72	1.66	1.60	1.76

The overall ROWAS II system was evaluated in passive mode, where the eight joints ran the closed-loop position control algorithms (section Closed-Loop Position Control for the ROWAS II System). Supported by the BWS system, the participant put the feet on the shoe platform (Figure 1B). The mechanisms of the bilateral shoulder, hip, knee, and ankle joints in the ROWAS II system were alighted to the corresponding joints of the participant. The participants passively followed the movement produced by the ROWAS II system and repeated each walking subsession three times. Please see the Supplementary Video “Test of the ROWAS II System” to see the performance of a representative participant during the experiment.

In the subtests of the ankle mechanism, the participant still used the overall ROWAS II system. The mechanisms of the bilateral shoulder, hip, and knee joints and the left ankle mechanism were fixed so that the participant was in a standing position. The right ankle mechanism ran firstly in passive mode, where the closed-loop position control algorithms were implemented. The participant supported their weight on their left foot, and followed the movement produced by the right ankle mechanism. Then the right ankle mechanism ran in active mode, where low admittance (*G*_*a*_ = 0.6) and high admittance (*G*_*a*_ = 1.6) gains were implemented. Four different movement cases were tested, which were:

(1) The participant always lifted up the right foot voluntarily during the whole gait cycle;
(2) The participant lifted up and pushed down the right foot voluntarily within 30–60% and 60–80% of the gait cycle, respectively;
(3) The participant pushed down and lifted up the right foot voluntarily within 30–60% and 60–80% of the gait cycle, respectively;
(4) The participant always pushed down the right foot voluntarily during the whole gait cycle.

The four cases tested the response of the ankle mechanism to active ankle dorsiflexion and plantarflexion, which are often used ankle functions in normal walking (Winter, 2009). The participants repeated each ankle movement case three times in the experiment. Please see the Supplementary Video “Test of the ROWAS II System” to see the performance of a representative participant during the experiment.

RMSE is the normal value to represent the track error (Rubio, 2018; Rubio et al., 2019a,b; Gao et al., 2020). Therefore, the tracking error of the passive and active control algorithms was based on the RMSE for each joint movement:

(23) $$\begin{array}{l} \theta_{pRMSE}=\sqrt{\frac{1}{N}\overset{N}{\underset{i=1}{\sum }}{\left(\theta_{ps}\left(i\right)-\theta \left(i\right)\right)}^{2}}, \end{array}$$

(24) $$\begin{array}{l} \theta_{aRMSE}=\sqrt{\frac{1}{N}\overset{N}{\underset{i=1}{\sum }}{\left(\theta_{as}\left(i\right)-\theta \left(i\right)\right)}^{2}}, \end{array}$$

where *N* is the number of data points during one gait cycle. θ_*ps*_ and θ_*as*_ were the simulated angle in passive and active modes, which were obtained using the simulated model (Figure 5).

## Results

### Evaluation of the ROWAS II System in Passive Mode

The closed-loop position control algorithms were implemented in the bilateral joint mechanisms of the ROWAS II system, but this paper only presented the results from the right side for sake of simplicity. After three tests were performed to the eight joints running in open loop, the steady-state gain for the shoulder (*k*_*s*_), hip (*k*_*h*_), knee (*k*_*k*_), and ankle (*k*_*a*_) mechanisms of the ROWAS II system were evaluated as:

(25) $$\begin{array}{l} k_{s}=-0.18;\;k_{h}=1.05;\;k_{k}=-0.27;\;k_{a}=0.60. \end{array}$$

**Figure 6:**
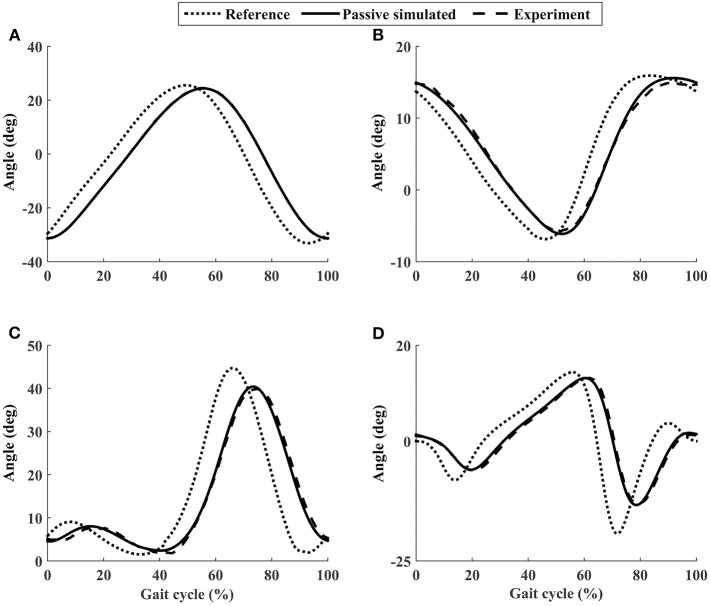
The joint performance of the representative participant P1 using the ROWAS II in passive mode. **(A)** Shoulder joint. **(B)** Hip joint. **(C)** Knee joint. **(D)** Ankle joint.

Using the closed-loop position control algorithms, the ROWAS II system produced synchronized walking with arm swing. A representative participant P1 (Figure 6) showed that ROMs of the shoulder, hip, knee, and ankle joints were in the normal range during human walking (Winter, 2009). The experimental values almost coincided with the simulated values with a RMSE of the shoulder, hip, knee, and ankle as 0.10°, 0.49°, 0.58°, and 0.41°, respectively. The ROWAS II system tracked the target trajectory well in passive mode, with θ_*pRMSE*_ of all joints for all participants <0.7° (from the second to the fifth rows in Table 2).

**Table 2:** RMSE values of all participants using the ROWAS II system running in passive mode.

**Test**	***θ_pRMSE_***	**P1**	**P2**	**P3**	**P4**	**P5**	**P6**
Overall ROWAS II	Shoulder (°)	0.10	0.10	0.10	0.10	0.10	0.10
	Hip (°)	0.49	0.33	0.28	0.40	0.35	0.34
	Knee (°)	0.58	0.48	0.50	0.59	0.52	0.54
	Ankle (°)	0.41	0.63	0.47	0.51	0.50	0.67
Ankle mechanism	Ankle (°)	0.45	0.42	0.40	0.46	0.45	0.46

### Evaluation of Active Ankle Control in the ROWAS II System

**Figure 7:**
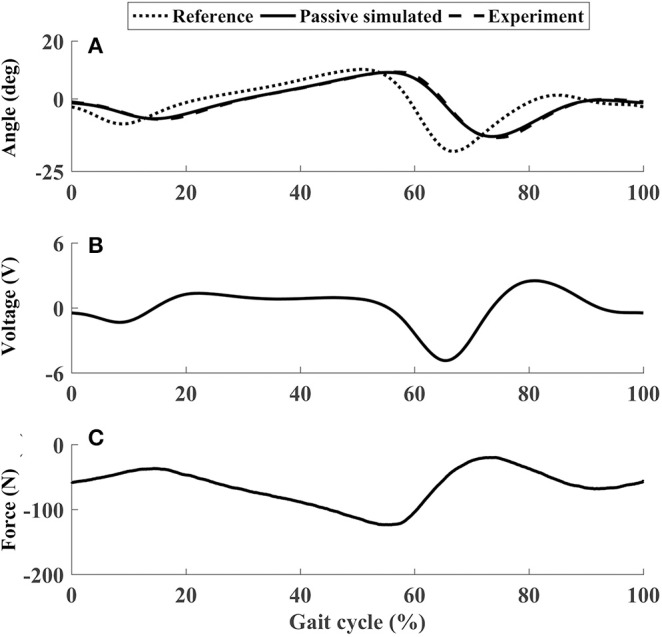
The performance of P1 using the ankle mechanism in passive mode. **(A)** Ankle joint trajectories. **(B)** Input voltage. **(C)** Passive force. In **(C)**, negative values mean that the force sensor was pulled.

In the passive subtest of the ankle mechanism, the force sensor measured the force required to achieve the target ankle movement (Figure 7C), which was important to calculate the voluntary input from the participant in the following active subtest. Figure 7B shows the voltage to the motor, which is proportional to the motor speed. In passive mode, the closed-loop position control algorithms enabled the ankle mechanism to track the target trajectories well (Figure 7A), with θ_*pRMSE*_ for all participants <0.5° (the sixth row in Table 2).

**Figure 8:**
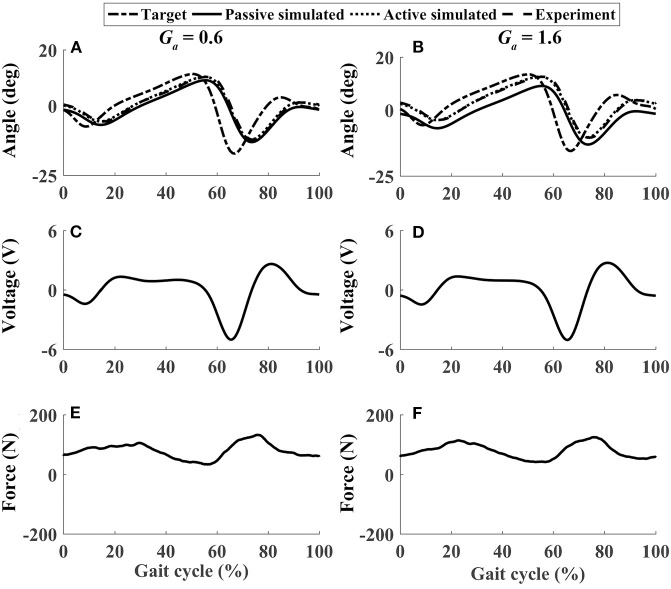
The performance of P1 when he lifted up his foot voluntarily during the whole gait cycle (Case 1). **(A,B)** Ankle joint trajectories. **(C,D)** Input voltage. **(E,F)** Active force.

**Figure 9:**
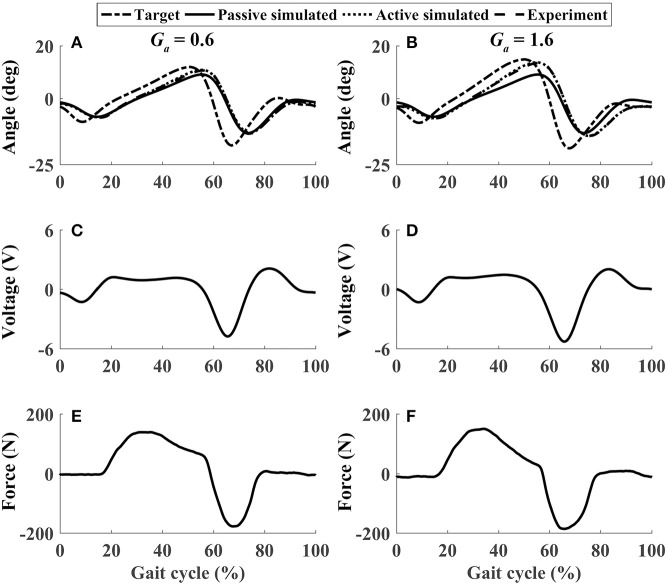
The performance of P1 when he lifted up and pushed down his foot voluntarily within 30–60% and 60–80% of the gait cycle, respectively (Case 2). **(A,B)** Ankle joint trajectories. **(C,D)** Input voltage. **(E,F)** Active force.

**Figure 10:**
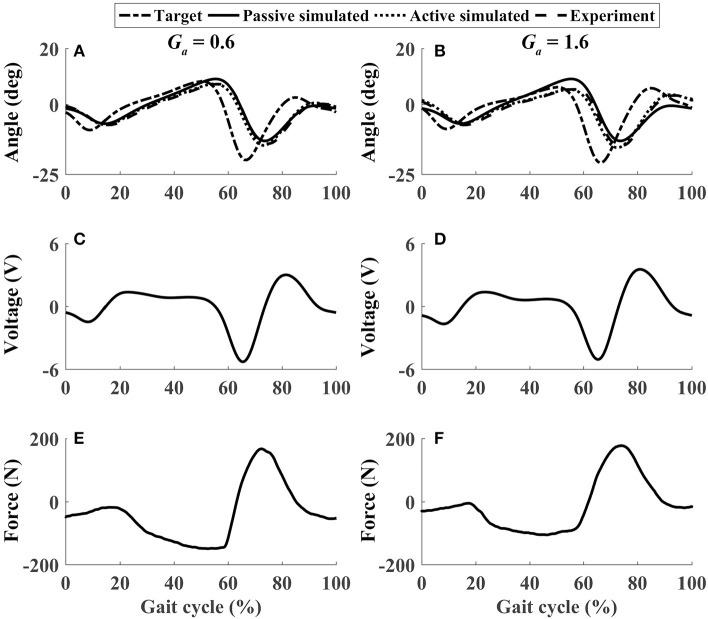
The performance of P1 when he pushed down and lifted up his foot voluntarily within 30–60% and 60–80% of the gait cycle, respectively (Case 3). **(A,B)** Ankle joint trajectories. **(C,D)** Input voltage. **(E,F)** Active force.

**Figure 11:**
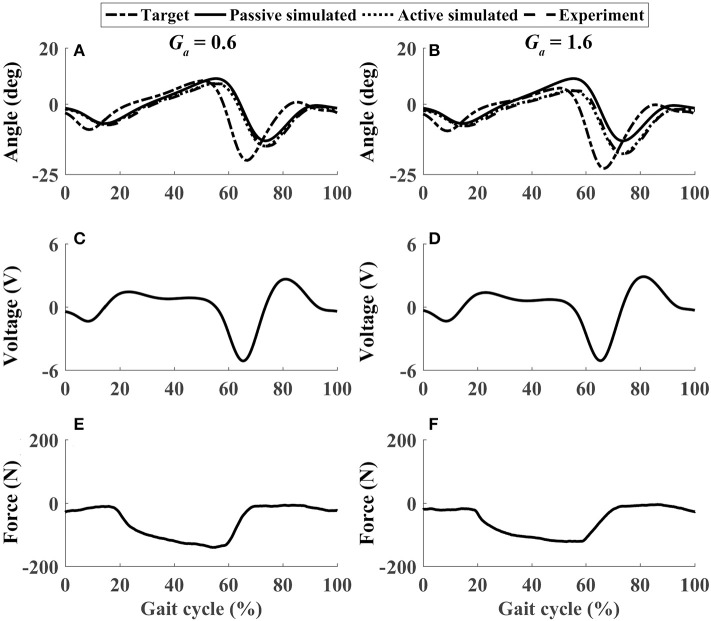
The performance of P1 when he pushed down his foot voluntarily during the whole gait cycle (Case 4). **(A,B)** Ankle joint trajectories. **(C,D)** Input voltage. **(E,F)** Active force.

In active mode, the participants finished four different experiment cases with the admittance gain *G*_*a*_ = 0.6 and *G*_*a*_ = 1.6, respectively. The results of P1 were presented in Figures 8–11, where the simulation result of passive movement was plotted to provide comparison. It was shown that P1 voluntarily adjusted the ankle movement. The active force *F* (Figures 8–11E,F) was obtained by subtracting the force measurement in passive mode (e.g., Figure 7C) from that recorded in active mode. The negative values of the active force mean that the participant pushed the foot downward. The more force P1 actively exerted to lift up the foot, the more upward movement the ankle mechanism generated (compared Figures 8A,E with Figures 9A,E, and compared Figures 8B,F with Figures 9B,F). This also applied when the participant pushed the foot downward. The more force P1 pushed down the foot, the more downward movement the ankle mechanism produced (compared Figures 10A,E with Figures 11A,E, and compared Figures 10B,F with Figures 11B,F). Besides, different admittance gains allowed the ankle mechanism to have different degrees of adjustment in response to active force. Compared with the experiments of the low admittance gain, P1 produced a significantly adjusted ROM of the ankle joint in the experiments of the high admittance gain [compared (A) with (B) in Figures 8–11, respectively].

**Figure 12:**
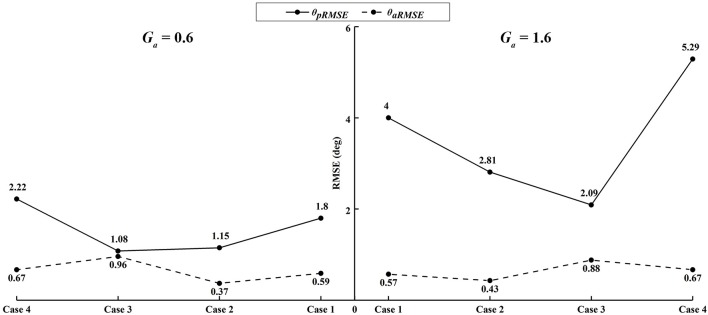
The average RMSE of all participants in four active ankle movement cases.

**Figure 13:**
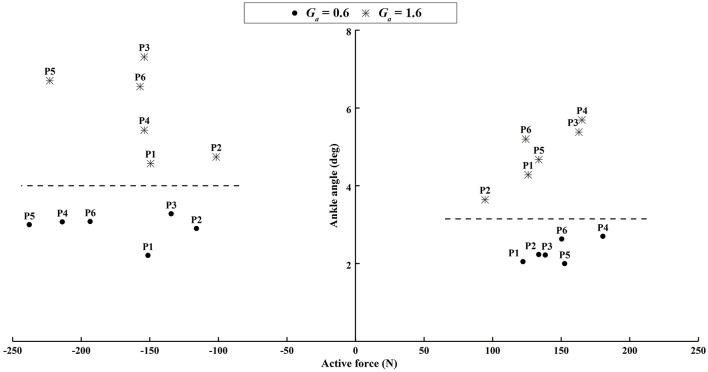
The difference of the peak ankle angle between the experimental and passive simulated results produced by different active force. The left subplot shows the results when the participants voluntarily pushed down the foot within 60–80% of the gait cycle. The right subplot shows the results when the participants voluntarily lifted up the ankle joint within 30–60% of the gait cycle.

Similar results were observed in all participants. The small errors between the experimental angle and the simulated active angle θ_*aRMSE*_ during the four active movement cases proved that the control algorithms yielded good tracking performance (θ_*aRMSE*_ < 1.0°, shown with dashed lines in Figure 12). The relatively large differences between the experimental angle and the simulated passive angle θ_*pRMSE*_ (solid lines in Figure 12) of all participants during the four active movement cases demonstrated that the participants voluntarily adjusted their ankle movement trajectories. No matter whether the participant lifted up or pushed down the foot, the ROM was more adjusted when the higher admittance gain was implemented (Figures 12, 13). Using the adjustable admittance control algorithms, all participants adjusted the ROM of the ankle mechanism by their voluntary input.

## Discussion

This work aimed to develop an improved rotational orthosis for walking with arm swing, and especially to develop and evaluate the algorithms for active ankle control. The closed-loop position control algorithms were implemented in the ROWAS II system in passive mode, while the adjustable admittance control algorithms were implemented in the ankle mechanism in active mode. Evaluated by six able-bodied participants, the ROWAS II system was technically feasible to produce walking-like movement in the bilateral upper and lower limbs in passive mode, and the ankle mechanism had technical potential to provide active training, where the ROM of the ankle joint was variously adjusted by the voluntary input from the users. The novel ROWAS II system can serve as a testbed for further investigation of interlimb neural coupling in response to voluntary ankle movement and is technically feasible to provide a new training paradigm of walking with arm swing and active ankle control.

The ROWAS II system differed from the previous systems (Fang et al., 2017a,b) in both mechanical structure and control system. The ROWAS and aROWAS systems had the rotary motors for the lower limbs mounted on bilateral sides. However, the ROWAS II system used series linear actuators which were installed behind the hip and knee mechanisms and in front of the ankle mechanism. This mechanical arrangement left the space around lateral hip joints free, therefore allowing natural arm swing. Furthermore, the force sensor at the end of the actuator provided the active force information, which enabled implementation of the adjustable admittance control algorithms, as demonstrated in the active control of the ankle mechanism in the ROWAS II system. The improved system ROWAS II provided more functions, which are discussed below.

The ROWAS II system produced synchronized arm-leg walking movement in passive mode. The steady gains of the shoulder, hip, knee, and ankle mechanisms were estimated using system identification. Based on the approximate gains, the closed-loop position control algorithms were developed using pole-placement approach. The control algorithms allowed the ROWAS II system to track the target trajectories very well. This demonstrated that the ROWAS II system produced walking-like movement with arm swing, and has technical potential to be applied in rehabilitation of walking for patients in the early post-injury stage.

Apart from passive training, the ROWAS II system achieved active training in the ankle mechanism after implementation of the adjustable admittance control algorithms. In active mode, the participants voluntarily performed four different ankle movements, which corresponded to the often used ankle function in normal walking (Winter, 2009). The relatively large difference between θ_*pRMSE*_ and θ_*aRMSE*_ (Figure 12) demonstrated that the participants voluntarily adjusted their ankle movement. The higher admittance gain enabled the participants to more easily change the movement trajectory of the ankle mechanism (Figures 12, 13). This novel ROWAS II system can serve as a testbed for further investigation of interlimb neural coupling in response to voluntary ankle movement and is technically feasible to provide a new training paradigm of walking with arm swing and active ankle control.

The limitation of this study was that only six participants were recruited. More participants are desirable to obtain a more detailed evaluation of the ROWAS II prototype. Nevertheless, six participants provided enough results for the technical evaluation of the system functionality. Furthermore, to allow an inter-individual comparison, a big screen should have been used so that the participant would have known how much active force was provided. Since the knee actuators also have force sensors, the adjustable admittance control algorithms will be implemented in both ankle and knee joints of the ROWAS II system. This study will be followed by the development of an overall active ROWAS II system and an investigation of the interlimb neural coupling in response to voluntary movement. Future work will apply the active ROWAS II system in clinical tests on patients with neurological impairments.

## Conclusions

The novel ROWAS II system was mechanically designed, manufactured and implemented with passive and active movement control algorithms. After evaluated by six able-bodied participants, the ROWAS II system produced synchronized walking movement with arm swing in passive mode. The adjustable admittance control algorithms enabled the ankle mechanism to be adjusted by the voluntary input from the user, with a higher admittance gain producing a larger degree of adjustment. The experimental evaluation demonstrated that the ROWAS II system and the control algorithms were technically feasible. The ROWAS II system has potential to serve as a testbed for further investigation of interlimb neural coupling in response to voluntary ankle movement and is technically feasible to provide a new training paradigm of walking with arm swing and active ankle control. After development of an overall active ROWAS II system, future work will focus on using the ROWAS II system to investigate the interlimb neural coupling in patients with neurological impairments.

## Data Availability Statement

The raw data supporting the conclusions of this article are available by the authors, without undue reservation, to any qualified researcher at https://drive.google.com/drive/folders/1_uUu4-yCYDK7bp5fQDxzHVjJtTFm9qdV?usp=sharing.

## Ethics Statement

The studies involving human participants were reviewed and approved by Med-X Research Institute, Shanghai Jiao Tong University, Shanghai, China (Ethical approval No. 2019051). The participants provided their written informed consent to participate in this study.

## Author Contributions

JF and ZM designed and manufactured the system, conducted the experiments, and analyzed the data. ZM drafted the manuscript. JF modified it critically. QZ supervised the experiments, analyzed the data, and checked this paper. G-YY and LX helped to provide the design concepts and checked this manuscript.

## Conflict of Interest

The authors declare that the research was conducted in the absence of any commercial or financial relationships that could be construed as a potential conflict of interest.

## Supplementary Material

Supplementary Video Test of the ROWAS II System.
